# Molecular Classification and Pharmacogenetics of Primary Plasma Cell Leukemia: An Initial Approach toward Precision Medicine

**DOI:** 10.3390/ijms160817514

**Published:** 2015-07-30

**Authors:** Vittorio Simeon, Katia Todoerti, Francesco La Rocca, Antonella Caivano, Stefania Trino, Marta Lionetti, Luca Agnelli, Luciana De Luca, Ilaria Laurenzana, Antonino Neri, Pellegrino Musto

**Affiliations:** 1 Laboratory of Pre-Clinical and Translational Research, Reference Cancer Center of Basilicata, Scientific Institute of Hospitalization and Treatment, Rionero in Vulture 85028, Italy; E-Mails: katiatodoerti@gmail.com (K.T.); francesco.larocca@crob.it (F.L.R.); caivanoa@libero.it (A.C.); stefania.trino@crob.it (S.T.); dr.luciana.deluca@gmail.com (L.D.L.); ilaria.laurenzana@crob.it (I.L.); 2 Department of Clinical Sciences and Community Health, University of Milan and Hematology, Fondazione Cà Granda, Ospedale Maggiore Policlinico, Milan 20122, Italy; E-Mails: marta.lionetti@unimi.it (M.L.); luca.agnelli@gmail.com (L.A.); antonino.neri@unimi.it (A.N.); 3 Scientific Direction, IRCCS-CROB, Referral Cancer Center of Basilicata, Rionero in Vulture 85028, Italy; E-Mail: pellegrino.musto@crob.it

**Keywords:** plasma cell leukemia, molecular profiling, risk stratification, pharmacogenetics, precision medicine

## Abstract

Primary plasma cell leukemia (pPCL) is a rare and aggressive variant of multiple myeloma (MM) which may represent a valid model for high-risk MM. This disease is associated with a very poor prognosis, and unfortunately, it has not significantly improved during the last three decades. New high-throughput technologies have allowed a better understanding of the molecular basis of this disease and moved toward risk stratification, providing insights for targeted therapy studies. This knowledge, added to the pharmacogenetic profile of new and old agents in the analysis of efficacy and safety, could contribute to help clinical decisions move toward a precision medicine and a better clinical outcome for these patients. In this review, we describe the available literature concerning the genomic characterization and pharmacogenetics of plasma cell leukemia (PCL).

## 1. Introduction

Plasma cell leukemia (PCL) is a rare and very aggressive variant of multiple myeloma (MM) defined by the presence of more than 20% of plasma cells (PC) in peripheral blood and/or an absolute PC count ≥2 × 10^9^/L [1,2]. In MM, malignant PCs are tightly dependent on the bone marrow microenvironment, which is essential for their growth and survival; however, myeloma cells increase their ability to spread to peripheral blood (PB) and extramedullary sites during the course of this disease [2]. This dissemination to PB is caused by different expression of adhesion molecules and chemokine receptors sustained by several molecular aberrations [3]. PCL is classified as primary (pPCL) when it presents *de novo* in patients with no evidence of previous MM, or secondary (sPCL) when it is observed as a leukemic transformation in relapsed or refractory MM patients [1]. The incidence of PCL in Europe is estimated around 0.04 cases per 100,000 persons per year, with a range of 2%–4% of patients with MM. Of this small percentage, 50%–70% of PCL are primary and the remaining are secondary forms [4,5,6].

pPCL patients show distinctive clinical-biological features if compared with MM or sPCL. They have a younger age at presentation and, compared to MM patients, are characterized by more frequent extramedullary disease and renal failure, higher bone marrow infiltration and proliferative activity of the malignant clone, and a low occurrence of bone disease [1,7]. Generally, pPCL may be initially sensitive to aggressive chemotherapy (especially to transplant procedures), but due to its aggressiveness, it almost invariably relapses early and, overall, clinical outcome is very poor. sPCL, on the other hand, represents the refractory end-stage of MM with survival measured in weeks [4,7].

In pPCL, multiple adverse genetic abnormalities are already present at the time of onset or diagnosis, whereas in sPCL they may gradually accumulate during progression from a previous MM phase, resulting in the acquisition of a more aggressive phenotype [8]. New high-throughput technologies have allowed a better understanding of the mechanisms underlying the biology of aggressive PC dyscrasias. As in many other cancers, biological information at diagnosis could be helpful for a prognostic risk stratification of this disease, which can guide clinicians in therapeutic decisions.

During the last years, immunomodulatory drugs (thalidomide and lenalidomide) and the proteasome inhibitor bortezomib have undoubtedly modified the therapeutic scenario of MM, improving response rate, progression-free survival (PFS) and overall survival (OS). These agents are progressively getting into the treatment of pPCL [7]. Due to the low incidence and prevalence of PCL, most information about the efficacy and safety of these drugs in pPCL, as well as clinical-biological and genomic data, come from isolated case reports and small retrospective studies [9]. Only one prospective study has been published until now and many of the genomic analyses in consideration during this review come from this prospective series [10].

Here, we discuss the genomic characteristics of pPCLs based on conventional approaches, such as karyotype and fluorescence *in situ* hybridization (FISH) analyses, and more recent, new high-throughput technologies such as single nucleotide polymorphism (SNP)-array, gene expression profiling (GEP), miRNA expression profiling, and whole exome sequencing (WES). We also summarize findings about the efficacy and safety variability related to genetic variations for old and new therapeutic regimens currently used for PCL treatment. We conclude that the development of biomarker discovery and improvement of pharmacogenomics knowledge by new genomic technologies may lead to a better clinical outcome of this rare and aggressive tumor.

## 2. Molecular Classification and Prognostic Risk Stratification

Genomic characterization from previously published retrospective series of PCLs, mainly based on conventional karyotyping, FISH, and array comparative genomic hybridization (aCGH) analyses, indicated a scenario of major genetic lesions that can overlap those found in MM, although PCLs have several specific features [4,11]. The percentage of PCL patients with an abnormal clone detectable by cytogenetic analysis is significantly higher than in MM [12,13]. From this data it is possible to infer that clonal PC from PCL, as said before, has a higher proliferative capacity and cell turnover when compared with MM [13].

Different data and percentages were reported in past retrospective studies, mainly due to the difficulty of recruiting a valid and representative number of patients with this rare disease. In the paper by Chiecchio *et al*. [13], the majority of pPCLs (7/10; 70%) were non-hyperdiploid (non-HRD), while a HRD karyotype was evidenced in the remaining pPCLs and in one out of two sPCLs. In the data reported by Tiedemann *et al*. [4], all investigated pPCLs were exclusively non-HRD, and HRD was found only in sPCLs. With regard to chromosomal translocations, involving the immunoglobulin heavy-chain locus (IGH@) at 14q32, they have been frequently found in pPCL and sPCL patients (82%–87%). Data from retrospective studies indicated that the Cyclin D1 (*CCND1*) gene, on locus 11q13, was mostly targeted in pPCL, while 14q32 rearrangements targeting 4p16.3 (Fibroblast growth factor receptor 3—*FGFR3* and Multiple Myeloma SET domain—*MMSET*) or 16q23 (*MAF*) occurred more frequently in sPCL [2,4].

In particular, Chang *et al*. [14] observed t(11;14) and t(4;14) chromosomal translocations in four (50%) and three (25%) pPCL patients, respectively. This is in agreement with data from Avet-Loiseau *et al*. [15], showing a higher frequency of 33% for t(11;14) in comparison to 13% for t(4;14), and Chiecchio *et al*. [13], who reported t(11;14) in 40% of pPCL patients and the absence of t(4;14). Furthermore, a different prognostic significance was associated with these chromosomal aberrations, with a better overall survival (OS) associated with t(11;14) reported by Avet-Loiseau *et al*. [15] but not by Chiecchio *et al*. [13], while Chang *et al*. [16] showed t(4;14) as an independent predictor for a poor OS.

Chang *et al*. [16] reported that 17p and 13q deletions were more frequent in PCL than in MM. Furthermore, the presence of del(17p) and del(13q) was associated with shorter OS [9]. *TP53* inactivation, in addition to 17p deletion, can be caused by functionally mono- or bi-allelic coding mutations. Moreover, inactivation of TP53 can also happen by overexpression of negative regulatory elements, such as mouse double minute 2 homolog (MDM2), or by decreased activity of CDKN2A (p14ARF), a negative regulator of MDM2 [17,18]. Damaging of *TP53* surveillance can induce genetic instability with the development of complex genetic abnormalities, and may be a prerequisite for dysregulation of oncogenes such as *RAS* and *MYC* [4].

1q gains and 1p losses are more frequent in PCL and both aberrations are strongly correlated. Importantly, 1p deletions, but not 1q gains, appear associated with a shorter survival [16].

Mutations of *K-RAS* or *N-RAS* (at codons 12, 13, or 61) have been reported in a retrospective series in 27% of pPCLs and 15% of sPCLs [4]. The prevalence of these mutations in sPCL was similar to that described in MM (21%) [1,19]. Rearrangements of *MYC* (as 3ʹ FISH break apart) were evidenced in 33% of pPCL and sPCL tumors, in addition to amplification or 5ʹ translocations in 8% and 17% of patients, respectively, and were found associated with poorer OS in pPCL [4]. Even in the study by Chiecchio *et al*. [13], highly variable structural and numerical alterations were found affecting *MYC*, leading to increasing levels of *MYC* transcript, particularly in relation to the type of genomic abnormality.

Finally, Usmani *et al*. [20] examined differences in transcriptional profiles of pPCL in comparison to non-pPCL cases, including MM, sPCL, and human myeloma cell lines. Data from this study suggest that pPCL samples represent a well-defined molecular entity that is distinguished from non-pPCL cases by a 203 gene-signature, involving transcripts mainly concerned in the lipid-metabolism pathway.

Despite the number of studies and the attempts to characterize this disease, a comprehensive analysis of genomic aberrations and the application of other “omics” technologies are still lacking in prospective series of pPCL. Recently, our groups have provided an extensive biological and molecular characterization of a panel of 23 pPCLs included in a Phase II prospective trial, aiming to evaluate the efficacy of novel biological drugs (lenalidomide in combination with dexamethasone) in the treatment of pPCL [10] (Table 1).

**Figure 1:**
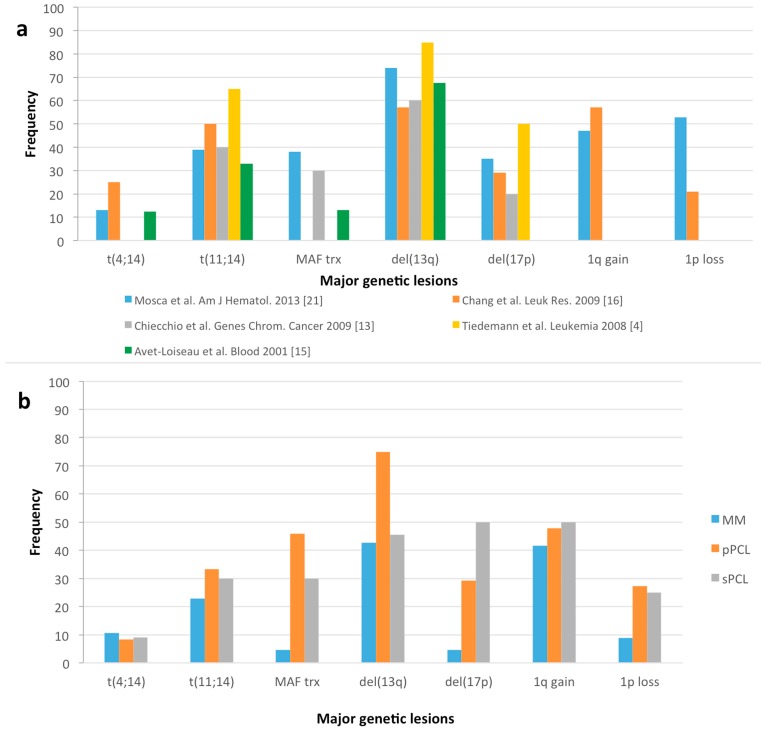
(**a**) Percentage of main genetic alterations in primary plasma cell leukemia (pPCL) patients of our prospective series compared to other retrospective pPCL cohorts; (**b**) Percentage of the same genetic lesions in the proprietary dataset of 132 multiple myeloma (MM) patients at onset, 24 pPCL and 11 secondary plasma cell leukemia (sPCL) cases (elaborated from Lionetti *et al*., Oncotarget 2015 [22]). Main chromosomal translocations, involving the immunoglobulin heavy-chain locus (IgH@) at 14q32 as t(4;14), t(11;14), MAF-translocations (trx), chromosome 13 and 17 deletions (del(13q), del(17p)), and chromosome 1 gains and losses (1q gain, 1p loss) are depicted.

Mosca *et al*. [21] provided a detailed genomic characterization of this prospective series by integrating SNP-array and FISH data with the corresponding transcriptome profiles. IGH@ translocations were identified in 87% of pPCL patients, with the most prevalence of t(11;14) (39%) and t(14;16) (30%). Meanwhile, the most frequent numerical alterations involved 1p (38%), 1q (48%), 6q (29%), 8p (42%), 13q (74%), 14q (71%), 16q (53%), and 17p (35%). They identified mutations of *TP53* in four cases, whereas activating mutations of the *BRAF* oncogene occurred in one case, while they were totally absent in N- and K-*RAS*. Furthermore, functional clustering analysis on differentially expressed genes mapped within altered copy number regions showed that the deregulated genes were mostly involved in processes fundamental for cancer development, such as intracellular protein transport, and Wnt and NF-kappa (NF-κB) pathways. In addition, several transcripts both in gained *(PSMB4*, *PSMD4*, *UCHL5*) and deleted (*PPP2CB*, *PPP2R5C*, *PSMC6*, *PSMD7*) chromosomal regions were associated with proteasomal ubiquitin-dependent protein catabolic process. Figure 1A shows the reported frequency of the major genetic alterations in our prospective pPCL series compared to other retrospective cohorts. As shown, the frequency of the main IGH@ translocations is variable in the different PCL series. For example, t(11;14) represents 39% of our population, which is close to that reported by Avet-Loiseau *et al*. [15] (33%) and Chiecchio *et al*. [13] (40%), but different from Tiedemann *et al*.’s series [4] (65%). *MAF* translocations were evidenced in 38% of pPCLs in our series, comparable to the 30% reported by Chiecchio *et al*. [13], whereas they were totally absent in Tiedemann *et al*.’s series [4]. Figure 1B shows the frequencies of the major genomic alterations in the prospective pPCL series compared to a proprietary retrospective cohort of newly diagnosed MM patients and sPCL cases [22]. As shown, the overall frequency of the major genetic alterations in PCL, in particular the 17p deletion, is higher than that found in MM.

**Table 1:** High-throughput technologies in pPCL classification.

Reference	pPCL Patients	Molecular Classification	Integrated Analysis	Gene/miRNA Signature (pPCL *vs.* MM)	Clinical Outcome Signature	Gene/miRNA List
Usmani *et al*., 2012 [20]	13	GEP	-	203 DE genes	-	-
Mosca L. *et al*., 2013 [21]	23	FISH	GEP	-	-	-
	17	SNP-array				
Todoerti *et al*., 2013 [23]	21	GEP	-	503 DE genes	3 genes-response rate	*YIPF6*, *EDEM3*, *YB5D2*
					27 genes-OS	*PECAM1*, *MKX*, *FAM111B*, *MCTP1*, *CALCRL*, *C10orf10*, *FNBP1*, *EFEMP1*, *C3orf14*, *ALDH1L2*, *WARS*, *SLC15A2*, *FAIM3*, *CPEB4*, *EDN1*, *PVALB*, *LY86*, *LAPTM5*, *RNU5D*, *PARP15*, *PLEKHF2*, *PDK4*, *TNFAIP3*, *FAM105A*, *CTH*, *HOOK1*, *TCN2*
Lionetti *et al*., 2013 [24]	18	miRNA	SNP-array/GEP	83 DE miRNAs	4 miRNAs-treatment response	miR-106b, miR-497, miR-181b, miR-181a
					4 miRNAs-PFS/OS	miR-92a, miR-330-3p, miR-22, miR-146a
Cifola *et al*., 2015 [25]	12	WES	SNP-array/GEP	-	-	-

DE—differentially expressed; GEP—gene expression profiling; FISH—fluorescence in situ hybridization; SNP—single nucleotide polymorphism; OS—overall survival; PFS—progression free survival; WES—whole exome sequencing.

Concerning the transcriptomic profiles, Todoerti *et al*. [23] investigated the gene expression profiles of 21 pPCL patients of the same series by means of microarray technology, correlating them with the primary and secondary outcome endpoints. As in MM, the main IGH@ chromosomal translocations drive the clustering of pPCL patients and are associated with specific transcriptional signatures. Interestingly, the combined analysis of 21 pPCLs and 55 newly diagnosed MM cases clustered together both PC dyscrasia samples in the main IGH@ translocation groups, thus suggesting that the presence of these chromosomal translocations, rather than the specific disease, strongly affects the transcriptional profiles in pPCL. Indeed, a 503-gene signature was able to distinguish pPCL from MM, highlighting the involvement of cytoskeleton functions, Rho protein signalling, and NF-κB pathways. Only a small overlapping (15%) with the GEP signature reported by Usmani *et al*. [20] in pPCL compared to non-pPCL samples was observed.

Furthermore, 26 genes of the identified transcriptional signature showed an expression trend associated with disease progression from normal PC condition throughout PCL. Finally, the expression levels of three genes (*CYB5D2*, *EDEM3*, and *YIPF6*) were correlated with response to the first-line treatment with lenalidomide/dexamethasone and a specific signature of 27 genes, resulting mostly up-regulated (17/27, 63%) in the pPCL patients with the poorest outcome, was associated with OS. Notably, the identified 27-gene model was independently associated with OS after adjustment for major cytogenetic alterations and hematological parameters. Importantly, the identified 27-gene signature retained significant correlation with outcome against known gene-risk models in MM, which stratify high- and low-risk myeloma patients on the basis of gene expression profile (UAMS 70-gene and 17-gene [26]; IFM 15-gene [27] and UK 6-gene [28]) [23]. On the other hand, the low-risk signature was found strongly associated with the autologous stem cell transplantation (ASCT) procedure and, consequently, with a more favorable prognosis.

MicroRNAs are short non-coding RNAs that control cell functions through mRNA targeting. Recently, abnormal expression of miRNAs has been reported in most of the solid or hematopoietic malignancies, including MM [29,30], and several preclinical findings have demonstrated their broad anti-cancer activity in the disease [31]. A global miRNA expression profiling was performed by microarray analysis in 18 pPCLs of the same series by Lionetti *et al*. [24]. A specific pattern of differentially regulated miRNAs (42 up-regulated and 41 down-regulated) was identified in pPCL when compared with a representative series of 39 MM. Interestingly, up-regulated miRNAs in pPCL were enriched in “onco-miRNAs”, such as miR-21 and miR-155, and recognized in miR-17~92 and miR-106a~363 clusters (miR-18a, miR-19a, miR-18b, and miR-20b). Interestingly, the expression of some miRNAs was associated with the DNA copy number of the corresponding loci, such as chromosomes 13 and 1. Finally, four miRNAs (miR-497, miR-106b, miR-181a, and miR-181b) were correlated with treatment response, and four (miR-92a, miR-330-3p, miR-22, and miR-146a) with clinical outcome. Among others, miR-146a and miR-22 deserve some attention. MiR-146a plays an important role in the regulation of innate immune and inflammatory responses through a negative feedback pathway involving NF-κB [32] and has been associated with the pathogenesis of several human diseases, whereas miR-22 was found significantly down-regulated in agreement with the allelic loss of its locus at 17p13.3 [33].

Finally, to provide knowledge about the mutational profile of this disease, Cifola *et al*. [25] performed WES analysis of 12 pPCL cases included in the same prospective trial. They identified 1928 coding somatic non-silent variants on a total 1643 genes, with a mean of 166 variants per sample. Interestingly, few variants and genes were recurrent in two or more samples. They were able to identify 14 candidate cancer driver genes involved in cell-matrix adhesion, cell cycle, genome stability, RNA metabolism, and protein folding. Furthermore, the integration of mutation data with the copy number alteration profiles revealed biallelically disrupted genes with tumor suppressor functions. Globally, cadherin/Wnt signaling, the extracellular matrix, and cell cycle checkpoints were the most affected functional pathways. Furthermore, Cifola *et al.* [25] reported a marked involvement of the *TP53* gene, with a recurrent implication of the *ATM* and *ATR* genes: two checkpoint kinases required for cell cycle arrest and DNA damage repair activation. The co-occurrence of mutations in different members of the DNA damage response, such as *TP53*, *ATM*, *ATR*, *CHEK2*, *CDC25A*, *CDKN1A*, *BRCA1*, may suggest a potential synergic role in deregulated DNA repair function in pPCL. This study represents the first WES analysis of pPCL and indicates a remarkable genetic heterogeneity of mutational patterns. Moreover, concerning the mutational status of genes recognized in the mitogen-activated protein kinase (MAPK) signaling, such as *BRAF*, *NRAS*, and *KRAS*, more recently, Lionetti *et al*. [22] have demonstrated, in a large panel of 167 patients representative of the different forms of PC dyscrasias (132 MM, 24 pPCL, and 11 sPCL), that mutations were found in 12% (BRAF), 23.9% (NRAS), and 29.3% (KRAS) of cases, respectively. Overall, mutations involving all three genes occurred in 63.6% of sPCL, 59.8% of MM, and 41.7% of pPCL cases [22].

## 3. Pharmacogenetics of New and Old Agents: Adverse Drug Reaction and Efficacy

Characterization of tumor genomics can help physicians to determine a patient’s disease and therefore the treatment choice. Nevertheless, the study of germline genetic variations, known as pharmacogenetics, can define kinetics and dynamics of cancer therapy pharmacology [34], establishing the variability of efficacy and safety and guiding physicians to the best drug and dosage.

Due to the complexity and rarity of PCL, information about efficacy and safety of therapy is poor and is mainly based on single case reports and small retrospective series with few patients. There is no pharmacogenetic information deriving directly from PCL patients; knowledge about pharmacology of old and new drugs used in treatment is based mostly on MM [35], which has a similar therapeutic regimen.

For this reason, we reviewed available information about new and old regimens used in PCL treatment. Terms used for the search on PubMed were “name of drug”, “multiple myeloma or plasma cell leukemia”, and “pharmacogenetics or pharmacogenomics or polymorphisms”. Furthermore, we analyzed information on “The Pharmacogenomics Knowledgebase” (PharmGKB) [36], a manually curated database of pharmacogenetic variation, looking for associations with other diseases. We discussed information about old and new drugs used in MM (Table 2) and reported in Supplementary Table S1 all the clinical information on other diseases derived from PharmGKB analyses.

**Table 2:** Germline genetic variations and efficacy/safety of old and new drugs used in the treatment of PCL and MM.

Drug	Gene	SNP	Alleles	Amino Acid Translation	Annotation	Reference
Melphalan	*ERCC2*	rs13181	T>G	Lys751Gln or K751Q	longer time to treatment failure	Vangsted *et al*., 2007 [37]
	*XRCC3*	rs861539	G>A	Thr241Met T241M		
	*ALDH2*	rs440	T>C	-	response to HDM	Dumontet *et al*., 2010 [38]
		rs4646777	G>A	-		
	*GSTT2*	rs1622002	G>A	Met139Ile		
	*BRCA1*	rs799917	C>G; C>T; C>A	Pro824Gln; Pro824Leu		
		rs4986850	G>A; G>T; G>C	Asp646Tyr; Asp646Asn		
	*CYP1A1*	rs1048943	T>G; T>C; T>A	Ile462Val; Ile462Leu; Ile462Phe	disease progression	
	*RAD51*	rs1801320	G>C	-		
	*PARP4*	rs13428	G>A; G>C	Gly1280Arg; Gly1280Cys		
	*ALDH2*	rs886205	A>G	-	OS	
	*CYP1A1*	rs1048943	T>G; T>C; T>A	Ile462Val; Ile462Leu; Ile462Phe		
	*BRCA1*	rs4986850	G>A; G>T; G>C	Asp646Tyr; Asp646Asn	severe mucositis after HDM	
	*CDKN1A*	rs1801270	C>A	Ser31Arg		
	*XRCC1*	rs25487	T>C	Gln399Arg		
	*SLC7A5*	rs4240803	G>A	-	gastrointestinal side effects	Giglia *et al*., 2014 [39]
	*IL-1β*	rs1143627	-31 C/T	-	OS	Vangsted *et al*., 2009 [40]
Vincristine	*GLI1*	rs2228224	G>A	Gly892Asp	early-onset peripheral neuropathy	Broyl *et al*., 2010 [41]
		rs2242578	G>C	-		
	*DPYD*	rs1413239	C>T	-	late-onset peripheral neuropathy	
	*ABCC1*	rs3887412	A>C	-		
	*GSTP1*	rs1695	A>G	Ile105Val	response after chemotherapy	Maggini *et al*., 2008 [42]
	*TYMS* or *ENOSF1*	rs2790	A>T; A>G	-		
		rs699517	C>T	-	response after SCT	
Dexamethasone	*MRD1* or *ABCB1*	rs2032582	A>T; A>C	Ser893Ala; Ser893;Thr	OS	Maggini *et al*., 2008 [43]
		rs1045642	A>T; A>G	Ile1145Ile		
	*GSTP1*	rs1695	A>G	Ile105Val	response after chemotherapy	Maggini *et al*., 2008 [42]
	*TYMS* or *ENOSF1*	rs2790	A>T; A>G	-		
		rs699517	C>T	-	response after SCT	
Bortezomib	*NFKB1*	rs28362491	ATTG>del	-	PFS	Varga *et al*., 2015 [44]
	*TRAF3*	rs11160707	G>A	-	PFS	Du *et al*., 2011 [45]
	*NFKB2*	rs12769316	G>A	-	OS	
	*PSD*	rs1056890	G>A	-	OS	
	*MRP1* or *ABCC1*	rs4148356	G>A	Arg723Gln	PFS-OS	Buda *et al*., 2010 [46]
	*CASP9*	rs2020895	A>G	-	early-onset peripheral neuropathy	Broyl *et al*., 2010 [41]
		rs2020903	G>A	-		
		rs4646034	T>C	-		
	*ALOX12*	rs1126667	A>G	Gln261Arg		
		rs434473	A>G	Asn322Ser		
	*IGF1R*	rs1879612	T>C	-		
	*ERCC4*	rs1799800	G>A	-	late-onset peripheral neuropathy	
		rs1799801	T>C	Ser835Ser		
	*ERCC3*	rs2276583	G>A	-		
	*PPARD*	rs2267668	G>A	-		
	*CTLA4*	rs4553808	A>G	-	peripheral neuropathy	Favis *et al*., 2011 [47]
	*CTSS*	rs12568757	G>A	-		
	*PSMB1*	rs1474642	A>G	-		
	*TCF4*	rs1261134	A>T	-		
	*DYNC1I1*	rs916758	A>G	-		
Thalidomide	*ABCA1*	rs363717	C>T	-	peripheral neuropathy	Johnson *et al*., 2011 [48]
	*ICAM1*	rs1799969	G>A	Gly241Arg		
	*PPARD*	rs2076169	A>G	-		
	*SERPINB2*	rs6103	C>G	Asn404Lys		
	*SLC12A6*	rs7164902	C>G; C>A	Leu144Leu		
	*ERCC1* or *CD3EAP*	rs735482	A>C	Lys259Thr	response rate	Cibeira *et al*., 2011 [49]
	*ERCC5*	rs17655	G>C	Asp1104His		
	*XRCC5*	rs1051685	A>G	-		
	*ERCC1* or *CD3EAP*	rs735482	A>C	Lys259Thr	OS	
	*XRCC5*	rs1051685	A>G	-		
	*GSTT1*	rs4630	G>A	-	peripheral neuropathy	
	*CDKN1A*	rs3829963	C>A	-	VTE	Almasi *et al*., 2011 [50]
	*TNF-alpha*	rs361525	G>A	-	PFS-OS	Du *et al*., 2010 [51]
	*CYP2C19*	Extensive metabolizers		-	OS	Li *et al*., 2007 [52]
Lenalidomide	*NFKB1*	rs3774968	A>G	-	VTE	Bagratuni *et al*., 2013 [53]

HDM—high dose melphalan; OS—overall survival; PFS—progression free survival; VTE—venous thromboembolism; SCT—stem cell transplantation.

### 3.1. Melphalan

Melphalan has been the first drug with proper efficacy in MM and it still remains a master drug in these patients, especially in the context of autologous stem cell transplantation (ASCT) [54]. Anyway, there are few data about predictive factors of response to high-dose chemotherapy with melphalan (HDM) in MM patients or association with its severe side effects, such as mucositis, diarrhea, and myelosuppression [55].

Polymorphisms of genes involved in drug metabolism, DNA repair, and apoptosis have been studied and associated with different profiles of toxicity/efficacy in MM patients. In particular, the combination of the polymorphisms rs13181 in *ERCC2* and rs861539 in *XRCC3* was associated with a longer time to treatment failure [37]. In the study by Dumontet *et al*. [38], polymorphisms in *ALDH2*, *GSTT2*, and *BRCA1* genes significantly influenced response to therapy; variants in *CYP1A1*, *RAD51*, and *PARP* were independently correlated with disease progression, whereas SNPs in *ALDH2* and *CYP1A1* were associated with death. Furthermore, polymorphisms in *BRCA1*, *CDKN1A*, and *XRCC1* were associated with a higher prevalence of severe side effect, particularly the occurrence of severe mucositis after HDM. Giglia *et al*. [39] conducted a study to investigate the association of melphalan therapy and gastrointestinal side effects. In particular, they investigated the principal mediators of melphalan uptake, the amino acid transporters *LAT1* and *LAT2*, encoded by the *SLC7A5* and *SLC7A8* genes, respectively. This study shows that a patient carrying rs4240803 in *SLC7A5* had a higher requirement of total parenteral nutrition use.

Interestingly, polymorphisms of genes coding for proinflammatory cytokines, suspected to play a role in the pathogenesis of MM, were investigated in 348 MM patients undergoing HDM treatment followed by ASCT. Patients carrying the rs1143627 allelic variant in *IL-1b* showed a significantly longer survival than wild-type homozygous patients [40].

### 3.2. Vincristine

Vincristine has been one of the most widely used and effective drugs in MM and PCL treatment, although the dose-limiting toxicity and an incomplete understanding of the pharmacokinetics and pharmacogenetics of vincristine may limit its current therapeutic potential. Induced peripheral neuropathy may be a dose-limiting toxicity. In early-onset, rs2228224 and rs2242578 SNPs in the *GLI1* gene appeared to be associated with peripheral neuropathy. Late-onset was associated with polymorphisms in genes involved in drug metabolism, such as rs1413239 in *DPYD* and rs3887412 in *ABCC1* [41].

Maggini *et al*. [42] studied the response to dexamethasone-doxorubicin-vincristine (DAV) therapy in MM patients associated with polymorphisms in genes of drug metabolism (*GSTP1*) and DNA synthesis (*TYMS*). These patients were treated with DAV followed by a conditioning regimen and ASCT. In particular, rs1695 in *GSTP1* and rs2790 in *TYMS* were significantly associated with a poor response following chemotherapy. Interestingly, an increased risk was observed in patients carrying a combined genotype. Moreover, patients with rs699517 in *TYMS* showed a significantly poorer response after ASCT.

### 3.3. Cyclophosphamide

Due to the complex metabolism, this drug has been associated with a large number of genes and processes, such as hepatic *CYP450* (*CYP2A6*, *2B6*, *2C8*, *2C9*, *2C19*, *3A4*, and *3A5*), NADPH-mediated oxidation by aldehyde dehydrogenases (*ALDH1A1* and *ALDH3A1*), and conjugation with glutathione by glutathione *S*-transferases such as *GSTA1*, *GSTM1*, *GSTP1*, and *GSTT1* [35,56].

### 3.4. Dexamethasone

Maggini *et al*. [43] evaluated the effect of diplotypes of *MDR1* (*ABCB1)* gene polymorphisms (rs1045642 and rs2032582 positions) on the clinical outcome of MM cases treated with the DAV regimen. These two single nucleotide polymorphisms are in strong linkage disequilibrium and the analyses showed that survival probability was lower in wild-type homozygous carriers for both SNPs (55%) than for variant carriers (in heterozygous or homozygous). As reported for vincristine, rs1695 in *GSTP1* and rs2790 in *TYMS* were significantly associated with a poor response following chemotherapy [42].

### 3.5. Bortezomib

The proteasome inhibitor bortezomib has shown multifactorial biological effects on both myeloma and microenvironment cells, such as suppression of adhesion molecule expression and inhibition of angiogenesis. The initial rationale to use bortezomib was its inhibitory effect on the NF-κB pathway, which is highly activated in MM [57]. Varga *et al*. [44] retrospectively analyzed the role of the rs28362491 *NFKB1* polymorphism, an insertion/deletion variant, on the survival of 295 MM patients treated with bortezomib. Interestingly, patients carrying homozygous insertion have a better progression-free survival from bortezomib treatment than patients with the insertion/deletion or homozygous deletion. Another study investigated 26 polymorphic sites of NF-κB family member genes (*IKB-α*, *NFKB2*, and *TRAF3*) in 83 MM patients. Notably, rs11160707 in the *TRAF3* gene was significantly associated with a better PFS; patients with rs12769316 in *NFKB2* had a superior OS, while rs1056890 was associated with an inferior OS. The authors concluded that at multivariate analysis, *TRAF3* rs11160707 was an independent favorable factor for PFS [45].

Vangsted *et al*. [58] conducted a study on 348 patients undergoing HDM and ASCT, showing that carrying one or two defective *CYP2D6* alleles can be predictive, albeit not statistically significant, of a trend towards a better time-to-next treatment. Even *MDR1* and *MRP1* genes*,* known as *ABCC1*, can modify the capacity to mediate drug resistance. Interestingly, Buda *et al*. [46] retrospectively evaluated the role of *MRP1* and *MDR1* on outcomes in relapsed and/or refractory MM patients in therapy with bortezomib and pegylated doxorubicin. The rs4148356 polymorphism in the *MRP1* gene was associated with a longer PFS and OS.

Bortezomib-induced peripheral neuropathy is a dose-limiting toxicity. This adverse event requires adjustment of therapy and can affect quality of life. The early onset of peripheral neuropathy in patients treated with bortezomib was significantly associated with SNPs in apoptosis genes, such as *CASP9*, *ALOX12*, and *IGF1R*; SNPs in inflammatory genes like *MBL2* and *PPARD*, and *ERCC3* and *ERCC4* DNA repair genes, were statistically associated with late-onset neuropathy [41]. Furthermore, genes involved in immune response (*CTLA4*-rs4553808 and *CTSS*-rs12568757), drug binding (*PSMB1*-rs1474642), or with a functional role in the nervous system (*TCF4*-rs1261134 and DYNC1I1-rs916758) were associated with bortezomib-induced peripheral neuropathy [47].

### 3.6. Thalidomide

Thalidomide has a complex mechanism of action that is not yet fully understood [59]. This drug undergoes a process of biotransformation, generating a multitude of metabolites and the genotype variation of the candidate genes involved in its metabolism can interfere with efficacy and safety during therapy. To identify genetic variations able to modulate and predict the risk of thalidomide-related peripheral neuropathy, Johnson *et al*. [48] analyzed samples from 1495 MM patients. They reported associations with SNPs in *ABCA1* (rs363717), *ICAM1* (rs1799969), *PPARD* (rs2076169), *SERPINB2* (rs6103), and *SLC12A6* (rs7164902) genes. These data are supported by the hypothesis that genes that have a role in inflammation and repairing mechanisms of the peripheral nervous system may influence the risk of developing peripheral neuropathy after treatment [60].

Cibeira *et al*. [49] examined polymorphisms in genes involved in multidrug resistance, drug metabolic pathways, DNA repair systems, and cytokines in 28 relapsed/refractory MM patients treated with thalidomide as a single agent. Patients carrying SNPs in *ERCC1* (rs735482), *ERCC5* (rs17655), or *XRCC5* (rs1051685) showed a higher response rate to therapy. Specifically, polymorphisms in *ERCC1* (rs735482) and *XRCC5* (rs1051685) were associated with longer OS. Indeed, patients showing rs4630 in *GSTT1* had a lower frequency of induced peripheral neuropathy. Additionally, Almasi *et al*. [50] analyzed the risk of venous thromboembolism (VTE), a well-known adverse event of thalidomide treatment, in 111 retrospective MM patients. Particularly, MM cases with rs3829963 in the *CDKN1A* gene had a higher frequency of VTE compared with the wild-type genotype. Du *et al*. [51] tested the impact of TNF-alpha promoter polymorphisms on the clinical outcome. They analyzed 98 MM patients treated with the thalidomide and dexamethasone regimen. The *TNF*-alpha rs361525 polymorphism was associated with better PFS and OS, confirming a previous independent study on 81 refractory MMs [61].

Furthermore, the role of *CYP2C19* polymorphisms (CYP2C19*2-rs4244285, CYP2C19*3-rs4986893) was investigated in 92 MM cases, 62 of which were treated with thalidomide and dexamethasone and 30 of which were treated with thalidomide combined with chemotherapy. CYP2C19 extensive metabolizers had a higher and statistically significant overall response rate when compared to poor metabolizer patients carrying the CYP2C19*2 or *3 variants [52].

### 3.7. Lenalidomide

The antiangiogenic and immunomodulatory properties of lenalidomide have been associated with notable efficacy in newly diagnosed and relapsed/refractory MM patients [62]. Unfortunately, lenalidomide is associated with a significant risk of VTE. Bagratuni *et al*. [53] analyzed 200 MM patients in therapy with lenalidomide-based regimens, showing that, in patients that received low-dose aspirin as prophylaxis, rs3774968 in the *NFKB1* gene was associated with an increased risk of VTE.

## 4. Conclusions

PCL represents a unique subset of patients with an aggressive clinical presentation, poor prognosis, shorter survival, and a different biologic background compared to MM [1]. Several studies demonstrated that the intensive treatment, introduction of novel agents such as lenalidomide and bortezomib, and bone marrow transplantation (allogenic and autologous stem cell) improved the survival of PCL patients [2,5,7]. Unfortunately, survival is still inferior if compared with the outcome in newly diagnosed MM patients, indicating the necessity for novel treatment strategies [3]. PCL patients should be considered primary beneficiaries for the newer novel agents, alone or in combination, such as second-generation proteasome inhibitors (Carfilzomib, Ixazomib), third-generation immunomodulatory drugs (Pomalidomide), and monoclonal antibodies (Elotuzumab, Daratumumab), as well as inhibitors of histone deacetylase, Akt, or mTOR [3,62,63,64,65].

Besides pharmacological research, improved genomic classification can help us in distinguishing groups of patients that may differ not only at the time of diagnosis but also in how they evolve or become resistant [66,67,68]. Data derived from the only study involving a pPCL prospective series [10,21,23,24] provided an in-depth scenario of genomic alterations in pPCL, although it should be taken into account that the difference between these data with others previously published [4,20] may be due to the relatively small number of cases and thus to the heterogeneity of all the series investigated, the geographical factors, or the methodology used to screen samples (see Figure 1). Furthermore, the identified transcriptional profile distinguishing pPCL from MM in the proprietary series showed only a small overlapping with the Usmani gene signature [20], probably due to different generation arrays or the molecular stratification of pPCL and MM/non-pPCL samples used in GEP comparisons.

We have recently proposed, in our prospective PCL cohort [23], a gene model related to clinical outcome; specifically, three genes were associated with response rate and 27 genes were associated with OS, retaining independence in OS prediction with respect to several known gene-risk models described in MM [23]. This finding requires more studies and consensus in order to define a reduced and powerful signature for prognostic stratification [69]. Similarly, Lionetti *et al*. [24] defined a miRNA signature highlighting the role of these small non-coding RNAs in pPCL, also as possible novel therapeutic targets. As for the gene signature, a larger prospective series is required to better understand the clinical significance of miRNAs.

Next-generation sequencing (NGS) might shed new light in the genetic complexity of pPCL. Cifola *et al*. [25] showed the involvement of a disparate set of genes and pathways useful as actionable targets. This first WES study on prospective pPCL patients can open new scenarios, improving the existing therapeutic opportunity.

Furthermore, molecular and genomic variability can have a great effect on drug efficacy and toxicity. Unfortunately, we still lack knowledge about the mode of action of many new drugs and how they interact with more than one target gene/protein [66,70]. Some of the genes, mentioned in pharmacogenetic studies, suggest the involvement of common pathways such as DNA damage response and nucleotide excision repair (*RAD51*, *BRCA1*, *ERCCs*, and *XRCCs* genes), Receptor Activator of Nuclear Factor kappa-B (RANK) RANK ligand (RANKL) signaling, apoptosis, and the NF-κB pathway (*TRAF3*, *NFKB2*, *ICAM1*, *IGF1R*, *NFKB1*, *CASP9*), together with genes concerned in cytochrome P450 and glutathione metabolism (see Supplementary Figure S1, performed using GeneMANIA) [71]. At present, there is little and controversial information about their clinical relevance. In addition, the mechanisms of their side effects are not fully understood. The success of a cancer therapy depends also on dose-limiting toxicity that can compromise the patient’s quality of life. The potentiality to stratify patient treatment according to genetic risk factors associated with adverse events is really important, but large prospective clinical studies are needed before the introduction in clinical practice [34,35,66,67]. It is well known that PCL has a poor prognosis, but the possibility of selecting patients with a more favorable outcome, thus avoiding them having adverse drug reactions, could be a great advantage.

The completion of the human genome project in 2003 and the use of “omics” technologies, such as microarray and NGS, has allowed medical research to progressively identify and improve the use of biomarkers in clinical practice. This process is intended to grow in parallel with our ability to understand genomic data and the improvement in “omics” technologies in terms of cost- and time-efficiency [72]. The ability to integrate these data with clinical information is crucial and will be one of the main purposes of precision medicine [72]. Up until now, many biomarkers failed in the process that led to success and to clinical implementation [73], mainly due to difficulty in independently replicating findings or in defining an exact event of adverse drug reaction. In a disease like PCL, with a small number of available cases, this risk becomes much more important.

Enrollment in large, collaborative, and well-organized clinical trials will thus become particularly important, expecting to have access to electronic healthcare and “omics” databases where patients can be followed longitudinally from the time of diagnosis to the clinical outcome [66,67,68]. Even as a “niche” disease with a set of problems with large enrollment and prospective validations, PCL could represent a valid model for precision medicine due to the necessity to decide on the treatment with the best efficacy/toxicity ratio for each patient as fast as possible.

## Supplementary Materials

Supplementary materials can be found at http://www.mdpi.com/1422-0067/16/08/17514/s1.
