# Supplementary material for: Molecular Classification and Pharmacogenetics of Primary Plasma Cell Leukemia: An Initial Approach toward Precision Medicine
Source: Int J Mol Sci. 2015 Jul 30;16(8):17514–34. doi: 10.3390/ijms160817514 (PMC4581206; doi:10.3390/ijms160817514)
Supplement: Supplementary file 1 [file ijms-16-17514-s001.pdf]

Supplementary Information

Supplementary Figure S1: Genes were selected through systematic search on PubMed database, looking for “name of drug”, “multiple myeloma or plasma cell leukemia”, and “pharmacogenetics or pharmacogenomics or polymorphisms”. GeneMANIA network analysis was performed on the global list of pharmacogenetic marker genes to reveal their potential associations, in terms of co-expression, co-localization, physical interactions, shared protein domains and pathways. Significant networks that were identified for all the listed genes (a) and for the ones specifically involved in the most representative pathways, DNA repair/response to DNA damage (b) and drug biotransformation (c), are reported.

Supplementary Table S1: Analysis of “The Pharmacogenomics Knowledgebase”, clinical information of germline genetic variation and efficacy/safety [1–45].

Table S1. Analysis of “The Pharmacogenomics Knowledgebase”, clinical information of germline genetic variation and efficacy/safety.

Drug	Gene	SNP	Alleles	Amico Acid Translation	Haplotypes	Annotation	Reference
Cyclophosphamide	*ABCB1*	rs10276036	C>T	-	ABCB1*13	Efficacy	Caronia *et al.*, 2011 [1]
		rs1128503	A>G	Gly412Gly	ABCB1*13	Efficacy	Caronia *et al.*, 2011 [1]
		rs2032582	A>T; A>C	Ser893Ala; Ser893Thr	ABCB1*13	Efficacy	Bray *et al.*, 2010 [2]
		rs4148737	T>C	-	-	Efficacy	Caronia *et al.*, 2011 [1]
	ABCC3	rs4148416	C>T	Gly1013Gly	-	Efficacy	Caronia *et al.*, 2011 [1]
	ABCC4	rs9561778	G>A; G>T	-	-	Toxicity/ADR	Low *et al.*, 2009 [3]
	ADH1C	rs698	T>C; T>A	Ile350Val	-	Efficacy	Khrunin *et al.*, 2014 [4]
	ALDH1A1	rs6151031	-	-	-	Toxicity/ADR	Ekhart *et al.*, 2008 [5]
	CYP2B6	CYP2B6 *1, CYP2B6 *6	-	-	-	Efficacy, Toxicity/ADR	Johnson *et al.*, 2013 [6]
		rs12721655	A>G	Lys139Glu	CYP2B6*8, CYP2B6*13, CYP2B6*13A, CYP2B6*13B	Efficacy	Bray *et al.*, 2010 [2]

		rs2279343	A>G	Lys262Arg	CYP2B6*4, CYP2B6*4A, CYP2B6*4B, CYP2B6*4C, CYP2B6*4D, CYP2B6*6, CYP2B6*6A, CYP2B6*6B, CYP2B6*6C, CYP2B6*7, CYP2B6*7A, CYP2B6*7B, CYP2B6*13, CYP2B6*13A, CYP2B6*13B, CYP2B6*16, CYP2B6*19, CYP2B6*20, CYP2B6*26, CYP2B6*34, CYP2B6*36, CYP2B6*37, CYP2B6*38	Toxicity/ADR	Rocha *et al.*, 2009 [7]
		rs3211371	C>T	Arg487Cys	CYP2B6*5, CYP2B6*5A, CYP2B6*5B, CYP2B6*5C, CYP2B6*7, CYP2B6*7A, CYP2B6*7B	Toxicity/ADR	Bray *et al.*, 2010 [2]

		rs3745274	G>T	Gln172His	CYP2B6*6, CYP2B6*6A, CYP2B6*6B, CYP2B6*6C, CYP2B6*7, CYP2B6*7A, CYP2B6*7B, CYP2B6*9, CYP2B6*13, CYP2B6*13A, CYP2B6*13B, CYP2B6*19, CYP2B6*20, CYP2B6*26, CYP2B6*34, CYP2B6*36, CYP2B6*37, CYP2B6*38	Toxicity/ADR; Dosage	Bray *et al.*, 2010 [2]; Rocha *et al.*, 2009 [7]
		rs8192709	C>T	Arg22Cys	CYP2B6*2, CYP2B6*2A, CYP2B6*2B, CYP2B6*10	Toxicity/ADR	Rocha *et al.*, 2009 [7]
	CYP2C19	rs4244285	G>A; G>C	Pro227Pro	CYP2C19*2, CYP2C19*2A, CYP2C19*2B, CYP2C19*2C, CYP2C19*2D, CYP2C19*2E, CYP2C19*2F, CYP2C19*2G, CYP2C19*2H, CYP2C19*2J	Efficacy; Toxicity/ADR	Bray *et al.*, 2010 [2]; Ngamjanyaporn *et al.*, 2011 [8]
	CYP2E1	rs2070676	G>C	-	CYP2E1*1B	Efficacy, Toxicity/ADR	Khrunin *et al.*, 2012 [9]
		rs6413432	T>A	-	-	Efficacy	Khrunin *et al.*, 2012 [9]
	CYP3A4	rs2740574	C>T	-	CYP3A4*1B, CYP3A4*15B, CYP3A4*23, CYP3A4*24	Toxicity/ADR	Su *et al.*, 2010 [10]
	EPHX1	rs1051740	T>C	Tyr113His	-	Toxicity/ADR	Khrunin *et al.*, 2014 [4]
	ERCC1	rs11615	A>G	Asn118Asn	-	Toxicity/ADR	Khrunin *et al.*, 2010 [11]; Khrunin *et al.*, 2012 [9]
	ERCC2	rs1799793	C>T	Asp288Asn	-	Efficacy, Toxicity/ADR	Khrunin *et al.*, 2010 [11]; Khrunin *et al.*, 2012 [9]

	GATA3	rs3824662	C>A	-	-	Efficacy	Perez-Andreu *et al.*, 2013 [12]
	GSTA1, *GSTA6P*	rs3957357	A>G	-	-	Toxicity/ADR	Khrunin *et al.*, 2010 [11]; Khrunin *et al.*, 2012 [9]
	GSTM3	rs1799735	C>CCT; C>-	-	-	Toxicity/ADR	Khrunin *et al.*, 2010 [11]; Khrunin *et al.*, 2012 [9]
	GSTP1	rs1695	A>G	Ile105Val	-	Efficacy	Khrunin *et al.*, 2010 [11]; Khrunin *et al.*, 2012 [9]
		rs1695	A>G	Ile105Val	-	Efficacy, Toxicity/ADR	Oliveira *et al.*, 2010 [13]; Zhang *et al.*, 2011 [14]
	LIG3	rs1052536	C>T	-	-	Toxicity/ADR	Khrunin *et al.*, 2014 [4]
	MTHFR	rs1801133	G>A	Ala140Val	-	Toxicity/ADR	Henríquez-Hernández *et al.*, 2010 [15]; Robien *et al.*, 2004 [16]; Patiño-García *et al.*, 2009 [17]
	MTR	rs1805087	A>G	Asp919Gly	-	Toxicity/ADR	Cui *et al.*, 2011 [18]; Patiño-García *et al.*, 2009 [17]
	MUTYH	rs3219484	C>T	Val22Met	-	Toxicity/ADR	Khrunin *et al.*, 2014 [4]

	NAT2	rs1801280	T>C	Ile114Thr	NAT2*5, NAT2*5A, NAT2*5B, NAT2*5C, NAT2*5D, NAT2*5E, NAT2*5F, NAT2*5G, NAT2*5H, NAT2*5I, NAT2*5J, NAT2*5K, NAT2*5L, NAT2*5M, NAT2*5N, NAT2*5O, NAT2*5P, NAT2*5Q, NAT2*5R, NAT2*5S, NAT2*5T, NAT2*5U, NAT2*5V, NAT2*14C, NAT2*14F	Toxicity/ADR	Khrunin *et al.*, 2014 [4]
	NOS3	rs1799983	T>G	Asp298Glu	-	Efficacy	Choi *et al.*, 2009 [19]
		rs2070744	C>T	-	-	Efficacy	Choi *et al.*, 2009 [19]
	NQO1	rs1800566	G>A	Pro149Ser	-	Efficacy	Fagerholm *et al.*, 2008 [20]; Jamieson *et al.*, 2011 [21]; Khrunin *et al.*, 2014 [4]; Kolesar *et al.*, 2002 [22]; Kolesar *et al.*, 2011 [23]; Siegel *et al.*, 1999 [24]; Siegel *et al.*, 2001 [25]; Smith *et al.*, 2001 [26]
	NQO2	rs1143684	C>T	Leu47Phe	-	Efficacy	Jamieson *et al.*, 2011 [21]
	RAD52	rs11226	G>A	-	-	Toxicity/ADR	Khrunin *et al.*, 2014 [4]
	SLC22A16	rs12210538	A>G	Met409Thr	-	Toxicity/ADR	Bray *et al.*, 2010 [2]
		rs6907567	A>G	Asn104Asn	-	Efficacy	Bray *et al.*, 2010 [2]
		rs723685	A>G	Val252Ala	-	Dosage	Bray *et al.*, 2010 [2]
	SOD2	rs4880	A>G	Val16Ala	-	Efficacy	Glynn *et al.*, 2009 [27]

	TP53	rs1042522	G>C	Pro33Arg	-	Efficacy, Toxicity/ADR	Henríquez-Hernández *et al.*, 2010 [15]; Huang *et al.*, 2008 [28]; Khrunin *et al.*, 2010 [11]; Khrunin *et al.*, 2012 [9]; Kim *et al.*, 2009 [29]
	TPMT	rs1142345	T>C	Tyr240Cys	TPMT*3A, TPMT*3C, TPMT*3D, TPMT*3E	Efficacy	Khrunin *et al.*, 2014 [4]
	VEGFA	rs2010963	C>G	-	-	Efficacy	Orlandi *et al.*, 2013 [30]
	XRCC1	rs25487	T>C	Gln399Arg	-	Efficacy, Toxicity/ADR	Khrunin *et al.*, 2010 [11]; Khrunin *et al.*, 2012 [9]
Dexamethasone	*GATA3*	rs3824662	C>A	-	-	Efficacy	Perez-Andreu *et al.*, 2013 [12]
Doxorubicin	*ABCB1*	rs10276036	C>T	-	ABCB1*13	Efficacy	Caronia *et al.*, 2011 [1]
		rs1045642	A>T; A>G	Ile1145Ile	ABCB1*13	Efficacy	Cizmarikova *et al.*, 2010 [31]; Giovannetti *et al.*, 2011 [32]; Gréen *et al.*, 2012 [33]; Kafka *et al.*, 2003 [34]; Lal *et al.*, 2008 [35]
		rs1128503	A>G	Gly412Gly	ABCB1*13	Efficacy	Caronia *et al.*, 2011 [1]
		rs2032582	A>T; A>C	Ser893Ala; Ser893Thr	ABCB1*13	Efficacy; Pharmacocynetic	Bray *et al.*, 2010 [2]; Lal *et al.*, 2008 [35]
		rs4148737	T>C	-	-	Efficacy	Caronia *et al.*, 2011 [1]
	ABCC1	rs45511401	G>T	Gly671Val	-	Toxicity/ADR	Wojnowski *et al.*, 2005 [36]
	ABCC2	rs17222723	T>A	Val1188Glu	-	Toxicity/ADR	Wojnowski *et al.*, 2005 [36]
		rs8187710	G>A	Cys1515Tyr	-	Toxicity/ADR	Wojnowski *et al.*, 2005 [36]
	ABCC3	rs4148416	C>T	Gly1013Gly	-	Efficacy	Caronia *et al.*, 2011 [1]
	ABCC4	rs9561778	G>A; G>T		-	Toxicity/ADR	Low *et al.*, 2009 [3]
	CBR1	rs20572	C>T	Ala209Ala	-	Dosage	Lal *et al.*, 2008 [37]
		rs9024	G>A	-	-	Dosage	Gonzalez-Covarrubias *et al.*, 2009 [38]; Lal *et al.*, 2008 [37]
	CBR3	rs8133052	G>A	Cys4Tyr	-	Efficacy, Toxicity/ADR	Fan *et al.*, 2008 [39]
	CYBA	rs4673	A>G	Tyr72His	-	Toxicity/ADR	Wojnowski *et al.*, 2005 [36]

	CYP2B6	rs12721655	A>G	Lys139Glu	CYP2B6*8, CYP2B6*13, CYP2B6*13A, CYP2B6*13B	Efficacy	Bray *et al.*, 2010 [2]
		rs3211371	C>T	Arg487Cys	CYP2B6*5, CYP2B6*5A, CYP2B6*5B, CYP2B6*5C, CYP2B6*7, CYP2B6*7A, CYP2B6*7B	Toxicity/ADR	Bray *et al.*, 2010 [2]
		rs3745274	G>T	Gln172His	CYP2B6*6, CYP2B6*6A, CYP2B6*6B, CYP2B6*6C, CYP2B6*7, CYP2B6*7A, CYP2B6*7B, CYP2B6*9, CYP2B6*13, CYP2B6*13A, CYP2B6*13B, CYP2B6*19, CYP2B6*20, CYP2B6*26, CYP2B6*34, CYP2B6*36, CYP2B6*37, CYP2B6*38	Dosage	Bray *et al.*, 2010 [2]
	CYP2C19	rs4244285	G>A; G>C	Pro227Pro	CYP2C19*2, CYP2C19*2A, CYP2C19*2B, CYP2C19*2C, CYP2C19*2D, CYP2C19*2E, CYP2C19*2F, CYP2C19*2G, CYP2C19*2H, CYP2C19*2J	Efficacy	Bray *et al.*, 2010 [2]
	GATA3	rs3824662	C>A	-	-	Efficacy	Perez-Andreu *et al.*, 2013 [12]
	GSTA1	rs3957357	A>G	-	-	Efficacy	Gelderblom *et al.*, 2014 [40]
	GSTM1	GSTM1 null		-	-	Efficacy, Toxicity/ADR	Altés *et al.*, 2013 [41]

	MTR	rs1805087	A>G	Asp919Gly	-	Toxicity/ADR	Patiño-García *et al.*, 2009 [17]; Cui *et al.*, 2011 [18]
	NCF4	rs1883112	G>A	-	-	Toxicity/ADR	Wojnowski *et al.*, 2005 [36]
	NOS3	rs1799983	T>G	Asp298Glu	-	Efficacy	Choi *et al.*, 2009 [19]
		rs2070744	C>T	-	-	Efficacy	Choi *et al.*, 2009 [19]
	NQO1	rs1800566	G>A	Pro149Ser	-	Efficacy	Fagerholm *et al.*, 2008 [20]; Jamieson *et al.*, 2011 [21]; Khrunin *et al.*, 2014 [4]; Kolesar *et al.*, 2002 [22]; Kolesar *et al.*, 2011 [23]; Siegel *et al.*, 1999 [24]; Siegel *et al.*, 2001 [25]; Smith *et al.*, 2001 [26]
	NQO2	rs1143684	C>T	Leu47Phe	-	Efficacy	Jamieson *et al.*, 2011 [21]
	RAC2	rs13058338	T>A	-	-	Toxicity/ADR	Wojnowski *et al.*, 2005 [36]
	SLC22A16	rs12210538	A>G	Met409Thr	-	Toxicity/ADR	Bray *et al.*, 2010 [2]
		rs6907567	A>G	Asn104Asn	-	Efficacy	Bray *et al.*, 2010 [2]
		rs714368	T>C	His49Arg	-	Other	Lal *et al.*, 2007 [42]
		rs723685	A>G	Val252Ala	-	Dosage	Bray *et al.*, 2010 [2]
Thalidomide	*ABCC6*	rs2238472	C>T	Arg1268Gln	-	toxicity	Deeken *et al.*, 2010 [43]
	ATP7A	rs2227291	G>C	Val767Leu	-	toxicity	Deeken *et al.*, 2010 [43]
	CHST3	rs12418	G>A	-	-	efficacy	Deeken *et al.*, 2010 [43]
		rs1871450	G>A	-	-	toxicity/toxicity	Deeken *et al.*, 2010 [43]
		rs4148943	C>T	-	-	efficacy	Deeken *et al.*, 2010 [43]
		rs4148945	C>T	-	-	efficacy/tocixity	Deeken *et al.*, 2010 [43]
		rs4148947	T>C	-	-	efficacy	Deeken *et al.*, 2010 [43]
		rs4148950	G>A	-	-	efficacy/toxicity	Deeken *et al.*, 2010 [43]
		rs730720	C>T	-	-	efficacy	Deeken *et al.*, 2010 [43]
	CYP4B1	rs4646487	C>T	Arg173Trp	CYP4B1*3, CYP4B1*6	toxicity	Deeken *et al.*, 2010 [43]

	NAT2	rs1799931	G>A	Gly286Glu	NAT2*5S, NAT2*6I, NAT2*6J, NAT2*6S, NAT2*6T, NAT2*7, NAT2*7A, NAT2*7B, NAT2*7C, NAT2*7D, NAT2*7E, NAT2*7F, NAT2*7G	toxicity	Deeken *et al.*, 2010 [43]
	PPARD	rs1883322	C>T	-	-	efficacy	Deeken *et al.*, 2010 [43]
		rs2016520	C>T	-	-	efficacy	Deeken *et al.*, 2010 [43]
		rs3734254	C>T	-	-	efficacy	Deeken *et al.*, 2010 [43]
		rs6922548	A>G	-	-	efficacy	Deeken *et al.*, 2010 [43]
		rs7769719	G>A	-	-	efficacy	Deeken *et al.*, 2010 [43]
	SLC10A2	rs2301159	G>A	-	-	toxicity	Deeken *et al.*, 2010 [43]
	SPG7	rs12960	G>A	Arg688Gln	-	toxicity	Deeken *et al.*, 2010 [43]
		rs2292954	A>G	Thr503Ala	-	toxicity	Deeken *et al.*, 2010 [43]
	SULT1C4	rs1402467	C>G	Asp5Glu	-	efficacy	Deeken *et al.*, 2010 [43]
Vincristine	*ABCB1*	rs10276036	C>T	-	ABCB1*13	Efficacy	Caronia *et al.*, 2011 [1]
		rs1045642	A>G	Ile1145Ile	ABCB1*13	Efficacy	Ceppi *et al.*, 2014 [44]
		rs1128503	A>G	Gly412Gly	ABCB1*13	Efficacy	Caronia *et al.*, 2011 [1]
		rs4148737	T>C	-	-	Efficacy	Caronia *et al.*, 2011 [1]
		rs4728709	G>A	-	-	Toxicity/ADR	Ceppi *et al.*, 2014 [44]
	ABCC3	rs4148416	C>T	Gly1013Gly	-	Efficacy	Caronia *et al.*, 2011 [1]
	ACTG1	rs1135989	G>A	Ala403Ala	-	Toxicity/ADR	Ceppi *et al.*, 2014 [44]
	CAPG	rs3770102	G>T	-	-	Toxicity/ADR	Ceppi *et al.*, 2014 [44]
	CEP72	rs924607	C>T	-	-	Toxicity/ADR	Diouf *et al.*, 2015 [45]
	GATA3	rs3824662	C>A	-	-	Efficacy	Perez-Andreu *et al.*, 2013 [12]
	MTR	rs1805087	A>G	Asp919Gly	-	Toxicity/ADR	Patiño-García *et al.*, 2009 [17]; Cui *et al.*, 2011 [18]

ADR—adverse drug reaction.

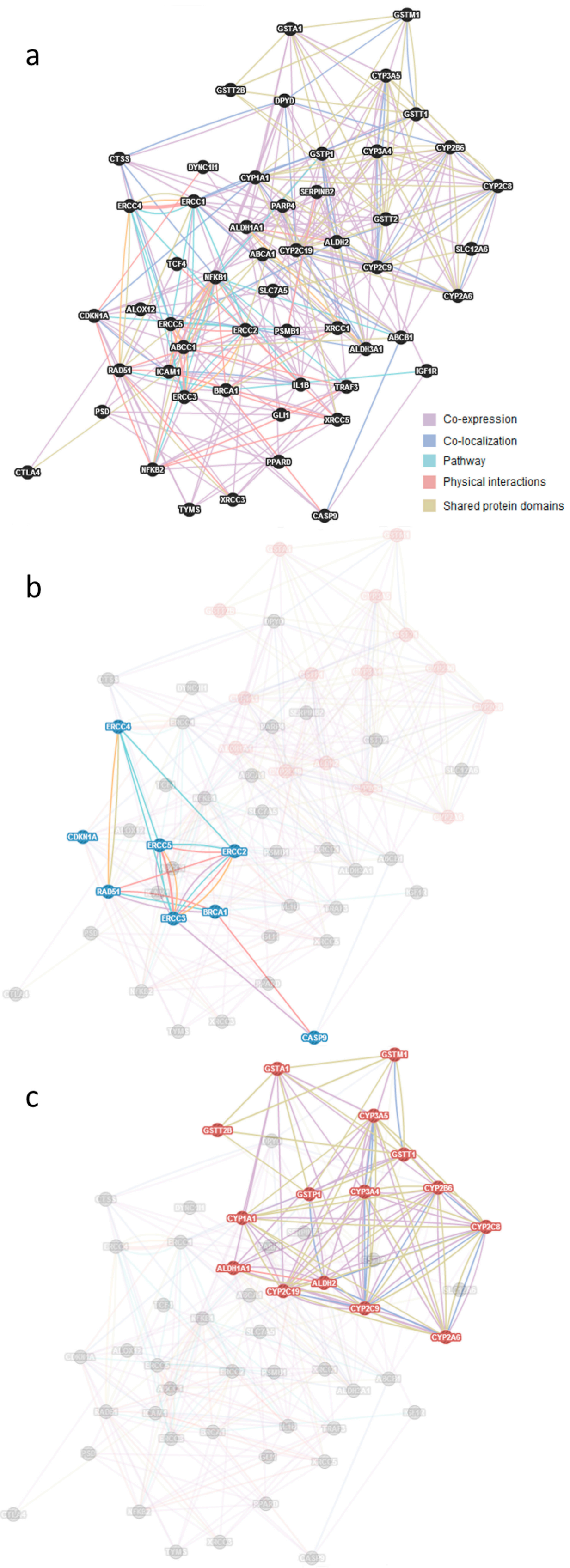

Figure S1. Gene networks of pharmacogenetic markers performed with GeneMANIA. (**a**) All listed genes; (**b**) genes involved in DNA repair/response to DNA damage; (**c**) genes involved in drug biotransformation.

References

1. Caronia, D.; Patiño-Garcia, A.; Peréz-Martínez, A.; Pita, G.; Moreno, L.T.; Zalacain-Díez, M.; Molina, B.; Colmenero, I.; Sierrasesúmaga, L.; Benítez, J.; *et al*. Effect of *ABCB1* and *ABCC3* polymorphisms on osteosarcoma survival after chemotherapy: A pharmacogenetic study. *PLoS ONE* **2011**, *6*, e26091.
2. Bray, J.; Sludden, J.; Griffin, M.J.; Cole, M.; Verrill, M.; Jamieson, D.; Boddy, A.V. Influence of pharmacogenetics on response and toxicity in breast cancer patients treated with doxorubicin and cyclophosphamide. *Br. J. Cancer* **2010**, *102*, 1003–1009.
3. Low, S.-K.; Kiyotani, K.; Mushiroda, T.; Daigo, Y.; Nakamura, Y.; Zembutsu, H. Association study of genetic polymorphism in *ABCC4* with cyclophosphamide-induced adverse drug reactions in breast cancer patients. *J. Hum. Genet.* **2009**, *54*, 564–571.
4. Khrunin, A.V.; Khokhrin, D.V.; Moisseev, A.A.; Gorbunova, V.A.; Limborska, S.A. Pharmacogenomic assessment of cisplatin-based chemotherapy outcomes in ovarian cancer. *Pharmacogenomics* **2014**, *15*, 329–337.
5. Ekhart, C.; Doodeman, V.D.; Rodenhuis, S.; Smits, P.H.M.; Beijnen, J.H.; Huitema, A.D.R. Influence of polymorphisms of drug metabolizing enzymes (*CYP2B6*, *CYP2C9*, *CYP2C19*, *CYP3A4*, *CYP3A5*, *GSTA1*, *GSTP1*, *ALDH1A1* and *ALDH3A1*) on the pharmacokinetics of cyclophosphamide and 4-hydroxycyclophosphamide. *Pharmacogenom. Genom.* **2008**, *18*, 515–523.
6. Johnson, G.G.; Lin, K.; Cox, T.F.; Oates, M.; Sibson, D.R.; Eccles, R.; Lloyd, B.; Gardiner, L.-J.; Carr, D.F.; Pirmohamed, M.; *et al. CYP2B6*6* is an independent determinant of inferior response to fludarabine plus cyclophosphamide in chronic lymphocytic leukemia. *Blood* **2013**, *122*, 4253–4258.
7. Rocha, V.; Porcher, R.; Fernandes, J.F.; Filion, A.; Bittencourt, H.; Silva, W.; Vilela, G.; Zanette, D.L.; Ferry, C.; Larghero, J.; *et al*. Association of drug metabolism gene polymorphisms with toxicities, graft-versus-host disease and survival after HLA-identical sibling hematopoietic stem cell transplantation for patients with leukemia. *Leukemia* **2009**, *23*, 545–556.
8. Ngamjanyaporn, P.; Thakkinstian, A.; Verasertniyom, O.; Chatchaipun, P.; Vanichapuntu, M.; Nantiruj, K.; Totemchokchyakarn, K.; Attia, J.; Janwityanujit, S. Pharmacogenetics of cyclophosphamide and *CYP2C19* polymorphism in Thai systemic lupus erythematosus. *Rheumatol. Int.* **2011**, *31*, 1215–1218.
9. Khrunin, A.; Ivanova, F.; Moisseev, A.; Khokhrin, D.; Sleptsova, Y.; Gorbunova, V.; Limborska, S. Pharmacogenomics of cisplatin-based chemotherapy in ovarian cancer patients of different ethnic origins. *Pharmacogenomics* **2012**, *13*, 171–178.
10. Su, H.I.; Sammel, M.D.; Velders, L.; Horn, M.; Stankiewicz, C.; Matro, J.; Gracia, C.R.; Green, J.; DeMichele, A. Association of cyclophosphamide drug-metabolizing enzyme polymorphisms and chemotherapy-related ovarian failure in breast cancer survivors. *Fertil. Steril.* **2010**, *94*, 645–654.
11. Khrunin, A.V.; Moisseev, A.; Gorbunova, V.; Limborska, S. Genetic polymorphisms and the efficacy and toxicity of cisplatin-based chemotherapy in ovarian cancer patients. *Pharmacogenom. J.* **2010**, *10*, 54–61.
12. Perez-Andreu, V.; Roberts, K.G.; Harvey, R.C.; Yang, W.; Cheng, C.; Pei, D.; Xu, H.; Gastier-Foster, J.; Lim, J.Y.-S.; Chen, I.-M.; *et al*. Inherited *GATA3* variants are associated with Ph-like childhood acute lymphoblastic leukemia and risk of relapse. *Nat. Genet.* **2013**, *45*, 1494–1498.

13. Oliveira, A.L.; Rodrigues, F.F.O.; Santos, R.E.; Aoki, T.; Rocha, M.N.; Longui, C.A.; Melo, M.B. *GSTT1*, *GSTM1*, and *GSTP1* polymorphisms and chemotherapy response in locally advanced breast cancer. *Genet. Mol. Res.* **2010**, *9*, 1045–1053.
14. Zhang, B.-L.; Sun, T.; Zhang, B.-N.; Zheng, S.; Lü, N.; Xu, B.-H.; Wang, X.; Chen, G.-J.; Yu, D.-K.; Lin, D.-X. Polymorphisms of *GSTP1* is associated with differences of chemotherapy response and toxicity in breast cancer. *Chin. Med. J.* **2011**, *124*, 199–204.
15. Henríquez-Hernández, L.A.; Murias-Rosales, A.; González-Hernández, A.; de León, A.C.; Díaz-Chico, N.; Fernández-Pérez, L. Distribution of TYMS, MTHFR, p53 and MDR1 gene polymorphisms in patients with breast cancer treated with neoadjuvant chemotherapy. *Cancer Epidemiol.* **2010**, *34*, 634–638.
16. Robien, K.; Schubert, M.M.; Bruemmer, B.; Lloid, M.E.; Potter, J.D.; Ulrich, C.M. Predictors of oral mucositis in patients receiving hematopoietic cell transplants for chronic myelogenous leukemia. *J. Clin. Oncol.* **2004**, *22*, 1268–1275.
17. Patiño-García, A.; Zalacaín, M.; Marrodán, L.; San-Julián, M.; Sierrasesúmaga, L. Methotrexate in pediatric osteosarcoma: Response and toxicity in relation to genetic polymorphisms and dihydrofolate reductase and reduced folate carrier 1 expression. *J. Pediatr.* **2009**, *154*, 688–693.
18. Cui, L.-H.; Yu, Z.; Zhang, T.-T.; Shin, M.-H.; Kim, H.-N.; Choi, J.-S. Influence of polymorphisms in *MTHFR* 677 C→T, *TYMS* 3R→2R and *MTR* 2756 A→G on NSCLC risk and response to platinum-based chemotherapy in advanced NSCLC. *Pharmacogenomics* **2011**, *12*, 797–808.
19. Choi, J.-Y.; Barlow, W.E.; Albain, K.S.; Hong, C.-C.; Blanco, J.G.; Livingston, R.B.; Davis, W.; Rae, J.M.; Yeh, I.-T.; Hutchins, L.F.; *et al.* Nitric oxide synthase variants and disease-free survival among treated and untreated breast cancer patients in a Southwest Oncology Group clinical trial. *Clin. Cancer Res.* **2009**, *15*, 5258–5266.
20. Fagerholm, R.; Hofstetter, B.; Tommiska, J.; Aaltonen, K.; Vrtel, R.; Syrjäkoski, K.; Kallioniemi, A.; Kilpivaara, O.; Mannermaa, A.; Kosma, V.-M.; *et al.* NAD(P)H:quinone oxidoreductase 1 *NQO1*2* genotype (P187S) is a strong prognostic and predictive factor in breast cancer. *Nat. Genet.* **2008**, *40*, 844–853.
21. Jamieson, D.; Cresti, N.; Bray, J.; Sludden, J.; Griffin, M.J.; Hawsawi, N.M.; Famie, E.; Mould, E.V.A.; Verrill, M.W.; May, F.E.B.; *et al.* Two minor *NQO1* and *NQO2* alleles predict poor response of breast cancer patients to adjuvant doxorubicin and cyclophosphamide therapy. *Pharmacogenom. Genom.* **2011**, *21*, 808–819.
22. Kolesar, J.M.; Pritchard, S.C.; Kerr, K.M.; Kim, K.; Nicolson, M.C.; McLeod, H. Evaluation of *NQO1* gene expression and variant allele in human NSCLC tumors and matched normal lung tissue. *Int. J. Oncol.* **2002**, *21*, 1119–1124.
23. Kolesar, J.M.; Dahlberg, S.E.; Marsh, S.; McLeod, H.L.; Johnson, D.H.; Keller, S.M.; Schiller, J.H. The *NQO1*2/*2* polymorphism is associated with poor overall survival in patients following resection of stages II and IIIa non-small cell lung cancer. *Oncol. Rep.* **2011**, *25*, 1765–1772.
24. Siegel, D.; McGuinness, S.M.; Winski, S.L.; Ross, D. Genotype-phenotype relationships in studies of a polymorphism in NAD(P)H:quinone oxidoreductase 1. *Pharmacogenetics* **1999**, *9*, 113–121.

25. Siegel, D.; Anwar, A.; Winski, S.L.; Kepa, J.K.; Zolman, K.L.; Ross, D. Rapid polyubiquitination and proteasomal degradation of a mutant form of NAD(P)H:quinone oxidoreductase 1. *Mol. Pharmacol.* **2001**, *59*, 263–268.
26. Smith, M.T.; Wang, Y.; Kane, E.; Rollinson, S.; Wiemels, J.L.; Roman, E.; Roddam, P.; Cartwright, R.; Morgan, G. Low NAD(P)H:quinone oxidoreductase 1 activity is associated with increased risk of acute leukemia in adults. *Blood* **2001**, *97*, 1422–1426.
27. Glynn, S.A.; Boersma, B.J.; Howe, T.M.; Edvardsen, H.; Geisler, S.B.; Goodman, J.E.; Ridnour, L.A.; Lønning, P.E.; Børresen-Dale, A.-L.; Naume, B.; *et al.* A mitochondrial target sequence polymorphism in manganese superoxide dismutase predicts inferior survival in breast cancer patients treated with cyclophosphamide. *Clin. Cancer Res.* **2009**, *15*, 4165–4173.
28. Huang, Z.-H.; Hua, D.; Li, L.-H.; Zhu, J.-D. Prognostic role of p53 codon 72 polymorphism in gastric cancer patients treated with fluorouracil-based adjuvant chemotherapy. *J. Cancer Res. Clin. Oncol.* **2008**, *134*, 1129–1134.
29. Kim, J.G.; Sohn, S.K.; Chae, Y.S.; Song, H.S.; Kwon, K.-Y.; Do, Y.R.; Kim, M.K.; Lee, K.H.; Hyun, M.S.; Lee, W.S.; *et al.* TP53 codon 72 polymorphism associated with prognosis in patients with advanced gastric cancer treated with paclitaxel and cisplatin. *Cancer Chemother. Pharmacol.* **2009**, *64*, 355–360.
30. Orlandi, P.; Fontana, A.; Fioravanti, A.; di Desidero, T.; Galli, L.; Derosa, L.; Canu, B.; Marconcini, R.; Biasco, E.; Solini, A.; *et al. VEGF-A* polymorphisms predict progression-free survival among advanced castration-resistant prostate cancer patients treated with metronomic cyclophosphamide. *Br. J. Cancer* **2013**, *109*, 957–964.
31. Cizmarikova, M.; Wagnerova, M.; Schonova, L.; Habalova, V.; Kohut, A.; Linkova, A.; Sarissky, M.; Mojzis, J.; Mirossay, L.; Mirossay, A. *MDR1* (C3435T) polymorphism: Relation to the risk of breast cancer and therapeutic outcome. *Pharmacogenom. J.* **2010**, *10*, 62–69.
32. Giovannetti, E.; Pacetti, P.; Reni, M.; Leon, L.G.; Mambrini, A.; Vasile, E.; Ghidini, M.; Funel, N.; Lucchesi, M.; Cereda, S.; *et al.* Association between DNA-repair polymorphisms and survival in pancreatic cancer patients treated with combination chemotherapy. *Pharmacogenomics* **2011**, *12*, 1641–1652.
33. Gréen, H.; Falk, I.J.; Lotfi, K.; Paul, E.; Hermansson, M.; Rosenquist, R.; Paul, C.; Nahi, H. Association of *ABCB1* polymorphisms with survival and *in vitro* cytotoxicty in *de novo* acute myeloid leukemia with normal karyotype. *Pharmacogenom. J.* **2012**, *12*, 111–118.
34. Kafka, A.; Sauer, G.; Jaeger, C.; Grundmann, R.; Kreienberg, R.; Zeillinger, R.; Deissler, H. Polymorphism C3435T of the *MDR-1* gene predicts response to preoperative chemotherapy in locally advanced breast cancer. *Int. J. Oncol.* **2003**, *22*, 1117–1121.
35. Lal, S.; Wong, Z.W.; Sandanaraj, E.; Xiang, X.; Ang, P.C.S.; Lee, E.J.D.; Chowbay, B. Influence of *ABCB1* and *ABCG2* polymorphisms on doxorubicin disposition in Asian breast cancer patients. *Cancer Sci.* **2008**, *99*, 816–823.
36. Wojnowski, L.; Kulle, B.; Schirmer, M.; Schlüter, G.; Schmidt, A.; Rosenberger, A.; Vonhof, S.; Bickeböller, H.; Toliat, M.R.; Suk, E.-K.; *et al.* NAD(P)H oxidase and multidrug resistance protein genetic polymorphisms are associated with doxorubicin-induced cardiotoxicity. *Circulation* **2005**, *112*, 3754–3762.

37. Lal, S.; Sandanaraj, E.; Wong, Z.W.; Ang, P.C.S.; Wong, N.S.; Lee, E.J.D.; Chowbay, B. *CBR1* and *CBR3* pharmacogenetics and their influence on doxorubicin disposition in Asian breast cancer patients. *Cancer Sci.* **2008**, *99*, 2045–2054.
38. Gonzalez-Covarrubias, V.; Zhang, J.; Kalabus, J.L.; Relling, M.V; Blanco, J.G. Pharmacogenetics of human carbonyl reductase 1 (*CBR1*) in livers from black and white donors. *Drug Metab. Dispos.* **2009**, *37*, 400–407.
39. Fan, L.; Goh, B.-C.; Wong, C.-I.; Sukri, N.; Lim, S.-E.; Tan, S.-H.; Guo, J.-Y.; Lim, R.; Yap, H.-L.; Khoo, Y.-M.; *et al*. Genotype of human carbonyl reductase *CBR3* correlates with doxorubicin disposition and toxicity. *Pharmacogenom. Genom.* **2008**, *18*, 621–631.
40. Gelderblom, H.; Blay, J.Y.; Seddon, B.M.; Leahy, M.; Ray-Coquard, I.; Sleijfer, S.; Kerst, J.M.; Rutkowski, P.; Bauer, S.; Ouali, M.; *et al*. Brostallicin versus doxorubicin as first-line chemotherapy in patients with advanced or metastatic soft tissue sarcoma: An European Organisation for Research and Treatment of Cancer Soft Tissue and Bone Sarcoma Group randomised phase II and pharmacogeneti. *Eur. J. Cancer* **2014**, *50*, 388–396.
41. Altés, A.; Paré, L.; Esquirol, A.; Xicoy, B.; Rámila, E.; Vicente, L.; López, R.; Orriols, J.; Vall-llovera, F.; Sánchez-González, B.; *et al*. Pharmacogenetic analysis in the treatment of Hodgkin lymphoma. *Leuk. Lymphoma* **2013**, *54*, 1706–1712.
42. Lal, S.; Wong, Z.W.; Jada, S.R.; Xiang, X.; Chen Shu, X.; Ang, P.C.S.; Figg, W.D.; Lee, E.J.; Chowbay, B. Novel *SLC22A16* polymorphisms and influence on doxorubicin pharmacokinetics in Asian breast cancer patients. *Pharmacogenomics* **2007**, *8*, 567–575.
43. Deeken, J.F.; Cormier, T.; Price, D.K.; Sissung, T.M.; Steinberg, S.M.; Tran, K.; Liewehr, D.J.; Dahut, W.L.; Miao, X.; Figg, W.D. A pharmacogenetic study of docetaxel and thalidomide in patients with castration-resistant prostate cancer using the DMET genotyping platform. *Pharmacogenom. J.* **2010**, *10*, 191–199.
44. Ceppi, F.; Langlois-Pelletier, C.; Gagné, V.; Rousseau, J.; Ciolino, C.; de Lorenzo, S.; Kevin, K.M.; Cijov, D.; Sallan, S.E.; Silverman, L.B.; *et al*. Polymorphisms of the vincristine pathway and response to treatment in children with childhood acute lymphoblastic leukemia. *Pharmacogenomics* **2014**, *15*, 1105–1116.
45. Diouf, B.; Crews, K.R.; Lew, G.; Pei, D.; Cheng, C.; Bao, J.; Zheng, J.J.; Yang, W.; Fan, Y.; Wheeler, H.E.; *et al*. Association of an inherited genetic variant with vincristine-related peripheral neuropathy in children with acute lymphoblastic leukemia. *JAMA* **2015**, *313*, 815–823.
